# When Suicide Is Not Suicide: Self-induced Morbidity and Mortality in the General Hospital

**DOI:** 10.5041/RMMJ.10197

**Published:** 2015-04-29

**Authors:** J. Michael Bostwick

**Affiliations:** Professor of Psychiatry, Mayo Clinic College of Medicine, Rochester, MN, USA

**Keywords:** Euthanasia, general hospital, hastened death, hospice, non-compliance, palliative care, suicide

## Abstract

Suicidal phenomena in the general hospital can take a variety of forms that can be parsed by taking into account whether or not the patient 1) intended to hasten death, and 2) included collaborators, including family and health care providers, in the decision to act. These two criteria can be used to distinguish entities as diverse as true suicide, non-compliance, euthanasia/physician-assisted suicide, and hospice/palliative care. Characterizing the nature of “suicide” events facilitates appropriate decision-making around management and disposition.

## INTRODUCTION

Suicide in the general hospital is hardly a unitary entity. Indeed the generic use of the term obscures the distinctions between several types of situations practitioners encounter that are called suicide but are best understood as something else. Entities lumped under the rubric of suicide range from patients who in attempts to end their lives have injured themselves severely enough to warrant admission for medical or surgical treatment, to those who are threatening to harm themselves for any number of reasons related to their hospitalizations, to those refusing or asking to withdraw from treatment, to those who through their non-compliant behavior are putting their lives at risk. Bostwick and Cohen have developed a model to assist in differentiating these phenomena by evaluating them according to two criteria: 1) Did the patient intend to hasten death or not? and 2) Did the patient involve the medical team and/or family and friends in the decision to hasten death?^1^ This article will discuss several permutations of criteria #1 and #2, proceeding from the premise that more clearly characterizing suicidal phenomena will result in more appropriate decision-making around management and disposition.

## TRUE SUICIDE

NM, a 34-year-old married woman, was videotaped pilfering small bills and change from the cash register at work. She was confronted with the evidence by her boss who told her that the police had been notified. Filled with shame, visibly distraught, she panicked. NM fled her workplace and drove straight home where she grabbed one of her husband’s hunting rifles with the intent of shooting herself in the head. When the gun bucked, the bullet shattered her humerus instead. She survived. Interviewed in the intensive care unit (ICU), NM said, “I really wanted to die and I still do. How will I face everyone when they learn what I’ve done?”

In true suicide, individuals desire immediate death. The acts they have committed and survived—or intend to commit—are potentially lethal, and they have taken no one else into confidence in discussing why death is a reasonable goal. The fact that they have not shared their plans or have not succeeded in killing themselves does not mean they are unambivalent about dying. As with NM, their actions are often impulsive. It is good clinical practice to assume that suicidal thinking or behavior in response to an acute event could prove—if effectively executed—a permanent solution to a temporary problem. Assessment can then focus on identifying remediable factors forcing the self-annihilatory fantasy or act.

Building on Gardner and Cowdry’s work,^2^ Jacobs and Bostwick have proposed eight scripts describing motives commonly underlying suicidal behavior. Among these motives are revenge, manipulation, shame, altruism, panic, and command hallucinations.^3^ Eliciting the narrative underlying the attempt or wish not only makes sense of what might otherwise appear senseless but also suggests interventions that could alleviate the suicidal urge.

Shneidman’s three-dimensional formulation of a suicidal crisis can aid in teasing out the contributors to a suicidal state.^4^ The three dimensions of his model are press, perturbation, and pain, dated terms that nonetheless correspond beautifully to contemporary stress-diathesis models.^5^ The first, *press*, is equivalent to *diathesis*, risk factors that by definition are longstanding, essentially immutable, indicative of general increased susceptibility. These include a personal or family history of affective illness, a family history of suicide, a personal history of previous attempts, active alcohol abuse or dependence, advanced age (particularly in men), and chronic, poorly managed pain. The second dimension, *perturbation*, is synonymous with *stress*, in the form of the recent onset of a noxious stimulus such as an acute depressive episode, acute pain, acute psychosis, acute loss of a significant relationship. The operative word is “acute,” superimposed upon the chronic backdrop implied in press. Finally, the third element is *pain*, a term encompassing the suicidal individual’s agonized mental state. Shneidman coined the word “psychache” to convey the unbearable emotional torment assailing suicidal individuals, a torment, a psychological torture they will do literally anything to escape.^6^ In his formulation, when press, perturbation, and pain are simultaneously operating at a fever pitch, their synergy yields a serious suicidal crisis.

Rudd makes the important observation that risk factors, by their nature static and enduring, afford little insight into whether a suicidal crisis is imminent. In line with Shneidman’s crisis model, he points out the fluid nature of intent and motivation for suicide, and calls for the assessment of warning signs indicating immediate risk. Warning signs are understood to relate to near-term danger—measured in minutes, hours, days—that rapidly evolve as the elements coalesce into an event, a suicide attempt.^7^,^8^ In NM’s case she has reacted to a potent trigger, an accusation of thievery, with instant panic. Her visible distress is a warning sign, as is her flight with only one intention: to lay her hands on the gun she needs to extinguish the shame and fear flooding her consciousness. Her tunnel vision is also a warning sign. It goaded her into seeing killing herself as the only possible alternative she had to her psychache.

It is critical to recognize that if the patient survives long enough, two of the three “p’s” can be mitigated, either through psychosocial or pharmacological interventions. Helping patients explore and understand what is behind their crises may aid them, both by giving voice to suicidal feelings that ebb when transformed into cognitions and by identifying problems that—once addressed—lose their power to fuel a deadly escape. For example, NM tells a sympathetic listener that she believes she will inevitably be incarcerated “for years, for decades for my crime.” Her distress dissipates upon learning that the police plan only to charge her with a misdemeanor, and the punishment for a first offender will likely be minimal.

While much is made of depression’s role in suicidal crises, definitive treatment of depressive episodes—if present—cannot be accomplished in the short term. However, judicious deployment of anti-psychotic or anxiolytic medication can nearly immediately mute the emotional upheaval of psychache. Likewise opiates or other analgesics may vanquish unbearable pain. Indeed medication administration may make it possible for an initially incoherent patient to become calm enough to tell their story.

In a case-control study, Shekunov and colleagues reviewed eight suicide attempts made by inpatients while hospitalized on Mayo Clinic medical or surgical units. They found cases differing from controls in that the former were more likely both to have psychiatric histories and to have had inpatient psychiatric consultation prior to the attempt. The agitation, impulsiveness, and disinhibition of delirium have long been known to raise the risk of self-injurious behavior in hospitalized patients.^9^ For hyperactive delirious states, close observation and neuroleptic medication can prevent quasi-suicidal acts. They also found that such remediable conditions as pain, anxiety, and insomnia were present in all attempters.^7^ They emphasized that attention to these issues in all patients constitutes both good medical practice and potential suicide prevention. Ultimately addressing what can be changed holds real promise of defusing the combustibility of a suicide crisis.

## NON-COMPLIANCE

RF, a 22-year-old man with a history of type-1 diabetes mellitus diagnosed at age five, is admitted with renal failure in the context of acute rejection of a transplanted kidney. Prior to receiving the kidney from a relative, he had frequently skipped dialysis sessions and refused to follow dietary guidelines for diabetics in renal failure. Non-compliance with anti-rejection medications precipitated the current rejection crisis. When embarking on a several-day binge of drinking and drugging, he had left his medications behind in his car which had been towed. The transplant team asked for guidance in managing RF’s “suicidal behavior.” “It’s not my fault that the cops took off with my car,” he said, when asked why he had failed to take his medications.

Another category of patients frequently labeled suicidal are the non-compliant. They engage in activities their caregivers realistically fear will damage them. Typically fragile health from an underlying medical condition requires them to live by more stringent rules than their non-afflicted peers. RF exemplifies such an individual, a diabetic refusing to adhere to dietary restrictions or his medication regimen, even when risking the loss of renal function, eyesight, or limbs to vascular complications of blood sugars run amok. Other examples are smokers who continue to light up despite worsening emphysema, or dialysis patients missing treatments because they have better things to do. According to the two principles for determining what constitutes true suicide, they neither wish—consciously—to hasten death nor collaborate with caregivers in making their self-destructive choices. While such patients may be literally killing themselves, the underlying problem is not a death wish but rather a refusal to submit to the limitations on normal life that their disease imposes. Approaches to treatment focus on behavior change, not suicide case management.

Similar to this group are the patients who engage in self-injurious behaviors, such as cutting their arms and legs, burning themselves, etc. Most psychiatrists recognize that these are parasuicidal behaviors in which the true intent is not to end life. Rather, such patients often have personality disorders or quasi-psychotic conditions, and they hurt themselves because of rage or as an attempt to avoid becoming psychotic. They carry out these behaviors without the complicity of their caregivers or loved ones.

## WITHDRAWAL OF LIFE SUPPORT TREATMENT

HF, an 80-year-old man with dialysis dependence in the context of worsening heart failure, has completed a cardiac work-up that concludes that nothing short of a heart transplant will allow him to regain the stamina required to take walks with his dog or play the golf he loves. Given this discouraging news, he asks to stop dialysis immediately. “This is not the life I want to live,” he says. The cardiology team requests a psychiatric consultation to evaluate his suicidality, given his abrupt decision with lethal implications.

Increasingly providers face patients asking for hastened death who desire that it take place with the collaboration of their chosen caregivers, including medical personnel, family, and friends.

Societal attitudes about what constitutes acceptable end-of-life choices have been evolving. In the early days of dialysis a half-century ago, candidates were carefully vetted for eligibility for what was an heroic treatment with limited availability. A 1971 article, still widely cited, claimed a suicide rate in dialysis patients of 5%, a number that seems inordinately high.^10^ A close reading of the article reveals that the vast majority of the “suicides” were actually deaths resulting from treatment non-compliance or those involving patients who discontinued treatment (without the approval of their physicians). The US Supreme Court ruling in Vacco vs. Quill differentiated between suicide and the death-accelerating practices of modern palliative medicine: treatment withdrawal, withholding, terminal sedation, and vigorous use of opiates. It underscored the right of patients autonomously to reach such decisions, and most of these situations are now reached through shared decision-making.^11^ Currently, among American patients receiving maintenance dialysis, at least a quarter of all deaths are preceded by these decisions.^12^ Hastened death is sought, and caregivers are actively involved.

## HOSPICE

RL, a 56-year-old diabetic man, lost one leg 14 months ago to an above-the-knee amputation (AKA) for gangrene stemming from peripheral vasculopathy. He is nearly blind with dialysis-dependent chronic renal failure and constant neuropathic pain in his remaining limbs. He now faces the same operation for identical pathology in his other leg. He feels his quality of life will be so impaired with bilateral AKAs and ongoing vascular insults that he refused the surgery and asks to be discharged to a nursing home and hospice care. “I don’t want any more cutting,” he says. “I just want to let nature take its course.”

RL has requested that his dialysis treatment be withdrawn, signaling a shift from aggressive curative care to palliation and symptom management. Over one million Americans with terminal illnesses die each year while receiving hospice services. Hospice principles seek to provide maximum symptom relief; to minimize hospitalizations, intensive care admissions, and surgeries; and to help dying individuals achieve their goals for the last phase of life. The hospice tries to involve family and friends and whenever possible to assist people to die at home rather than in institutions. Hospice and palliative care are highly collaborative, seeking to facilitate the dying individual and the caregivers in formulating common goals and making mutual decisions in order to achieve the best possible death.^13^

The Hospice Movement and palliative care specialists explicitly have *no* intention of speeding terminal patients toward early deaths. They *do* seek to alleviate suffering and *do* recognize that ministrations on behalf of dying individuals in excruciating pain—typically high-dose opiates—may contribute secondarily to fatal respiratory suppression. The contradiction between relieving suffering and hastening death is reified in the ethical Principle of Double Effect, attributed to the thirteenth-century St Thomas Aquinas.^14^ If the caregiver’s actions are motivated primarily by the wish to do good—to relieve pain and suffering—then the secondary consequence of respiratory suppression unto death is acceptable and excusable.

## PHYSICIAN-ASSISTED SUICIDE (DEATH WITH DIGNITY)

Several US states and European countries have legalized physician-assisted suicide with guidelines defining which patients are eligible for limited assistance in dying.^10^,^11^ In the United States, this involves the physician providing a prescription for a lethal dose of barbiturates after the patient has completed a series of consultations meant to establish the terminal nature of their condition and their state of mind in seeking to hasten death. The patient must then wait a specific period of time and be able personally to administer the killing dose. Death with Dignity is the term used in Oregon, which has accumulated 17 years of data about patients who have relied on its law. The best known has been Brittany Maynard, the 29-year-old woman with a glioblastoma, who uprooted her life in California and moved with her family to Oregon so that she could avail herself of that state’s physician-assisted suicide statute. She died in November 2014 with her husband and parents at her bedside.^15^

Laws like Oregon’s are usually opposed by such physician organizations as the American Medical Association, which specifically objects to physicians actively assisting in hastening death.^16^ Palliative care specialists, supported by the 1997 Vacco vs. Quill ruling, find an intermediate stance in which palliative sedation and withdrawal of life-sustaining treatments are acceptable as long as the primary purpose is to reduce suffering as life ebbs.^17^,^18^

Given the highly collaborative nature of deaths like that of Maynard, who had the support of her family and the assistance of her physicians in hastening death, these clearly do not fit the traditional definition of suicide. And yet whether society should more broadly legitimate this way of dying remains a subject of debate.

## SUMMARY

This article’s intent is to make the case that many general hospital phenomena labeled “suicide” or “suicidal” are actually neither but rather represent a plethora of other entities, each with its own set of approaches to diagnosis and management. A two-question model clarifying whether or not hastened death is intended and whether or not collaborators are participating permits distinctions between concepts as diverse as true suicide, non-compliance, physician-assisted suicide, and palliative care of the terminally ill. Seeking the script—the narrative underpinning a suicidal crisis—can make sense of the suicidality while also suggesting psychosocial and pharmacologic means for defusing the lethal urge. In sum, simplistic or reductionistic uses of the term “suicide” serve neither patients nor caregivers well.
